# Lipoprotein Lipase S447X variant associated with VLDL, LDL and HDL diameter clustering in the MetS

**DOI:** 10.1186/1476-511X-10-143

**Published:** 2011-08-19

**Authors:** Alexis C Wood, Stephen Glasser, W Timothy Garvey, Edmond K Kabagambe, Ingrid B Borecki, Hemant K Tiwari, Michael Y Tsai, Paul N Hopkins, Jose M Ordovas, Donna K Arnett

**Affiliations:** 1 Department of Epidemiology, University of Alabama at Birmingham, School of Public Health, Alabama, USA; 2 Department of Biostatistics, Section on Statistical Genetics, University of Alabama at Birmingham, School of Public Health, Alabama, USA; 3 Department of Medicine, Division of Preventive Medicine, University of Alabama at Birmingham, Alabama, USA; 4 Birmingham Veterans Affairs Medical Center, Birmingham, Alabama, USA; 5 Nutrition Obesity Research Center, University of Alabama at Birmingham, School of Public Health, Alabama, USA; 6 Division of Statistical Genomics, Department of Genetics, Washington University, School of Medicine, 4444 Forest Park Boulevard - Box 8506, St. Louis, Missouri, USA; 7 Department of Laboratory Medicine and Pathology, University of Minnesota, MN, USA; 8 Department of Internal Medicine, University of Utah, Salt Lake City, Utah, USA; 9 Jean Mayer United States Department of Agriculture Human Nutrition Research Center on Aging, Tufts University, Boston, Massachusetts, USA; 10 The Department of Epidemiology and Population Genetics. Centro Nacional Investigación Cardiovasculares Madrid, Spain

**Keywords:** GOLDN, lipoprotein, lipoprotein lipase, Metabolic syndrome

## Abstract

**Background:**

Previous analysis clustered 1,238 individuals from the general population Genetics of Lipid Lowering Drugs Network (GOLDN) study by the size of their fasting very low-density, low-density and high-density lipoproteins (VLDL, LDL, HDL) using latent class analysis. From two of the eight identified groups (N = 251), ~75% of individuals met Adult Treatment Panel III criteria for the metabolic syndrome (MetS). Both showed small LDL diameter (mean = 19.9 nm); however, group 1 (N = 200) had medium VLDL diameter (mean = 53.1 nm) while group 2 had very large VLDL diameter (mean = 65.74 nm). Group 2 additionally showed significantly more insulin resistance (IR), and accompanying higher waist circumference and fasting glucose and triglycerides (all *P *< .01). Since lipoprotein lipase hydrolyzes triglyceride in the VLDL-LDL cascade, we examined whether these two patterns of lipoprotein diameter were associated with differences across two lipoprotein lipase (*LPL*) gene variants: D9N (rs1801177) and S447X (rs328).

**Findings:**

Mixed linear models that controlled for age, sex, center of data collection, and family pedigree revealed no differences between the two groups for the D9N polymorphism (*P *= .36). However, group 2 contained significantly more carriers (25%) of the 447X variant than group 1 (14%; *P *= .04).

**Conclusions:**

This was the first study this kind to show an association between *LPL *and large VLDL particle size within the MetS, a pattern associated with higher IR. Future work should extend this to larger samples to confirm these findings, and examine the long term outcomes of those with this lipoprotein diameter pattern.

## Findings

The MetS is characterized by three or more of the following features: increased waist circumference (WC), high triglycerides (TGs), lowered high density cholesterol (HDL-C), hypertension and impaired fasting glucose [1]. Shifts in overall lipoprotein diameter to smaller LDL and HDL particles, and to larger VLDL particles have additionally been shown to associate with the individual features of the MetS [2].

Our previous research grouped individuals according to the fasting diameters of their VLDL, LDL and HDL particles. Two groups emerged where individuals, on average, met the adult treatment panel (ATP) III criteria for the MetS. Both groups had small LDL diameters (mean = 19.9 nm), but one group with larger VLDL diameters (65.74 vs 53.1 nm) showed higher IR as measured by homeostatic model assessment (HOMA-IR; Table 1); and increased features of the MetS associated with higher IR: higher fasting glucose, TG, and WC (Table 1).

**Table 1:** Lipid, demographic, genetic and MetS-related characteristics for participants by group

	Group 1	Group 2
N	200	51
Gender; % male	72	61
Age, y	50.51 (14.99)	50.94 (14.84)
**Lipoprotein diameters**		
VLDL**	53.07 (3.33)	65.74 (4.92)
LDL	19.79 (0.43)	20.05 (0.56)
HDL	8.39 (0.23)	8.45 (0.24)
**Sera lipoprotein particle concentrations (nmol/L)**		
VLDL	108.62 (51.43)	76.43 (54.20)
LDL	1760.02 (469.19)	1624.28 (488.76)
HDL	29.06 (5.49)	30.22 (5.27)
**MetS, and related features**		
Waist circumference; cm	106.54 (14.80)	109.27 (14.26)
Fasting glucose, (mmol/L)**	5.99 (1.19)	6.37 (1.70)
Diabetes, %**	13	24
Fasting TGs (mmol/L)**	2.37 (1.07)	3.43 (3.76)
HOMA-IR*	4.92 (3.08)	6.56 (4.78)
Total No of MetS counts	3.44 (1.19)	3.45 (1.15)
Individuals meeting ATP-III MetS criteria; %	72.5	76.5
**Genotype data, Minor allele carriers, %**		
*LPL *S447X*	14	25
D9N	7	4

*P < .05 **P < .001 All models control for age, sex, centre of data collection and family pedigree.

*LPL *hydrolyzes TGs from VLDL forming LDL, thus is a source of lipoprotein size heterogeneity. We therefore aimed to examine whether there were differences in two variants in the *LPL *gene between two groups of MetS individuals, defined by their fasting lipoprotein diameter pattern.

## Materials and Methods

### Participants and study design

The study sample includes 251 men and women participating in the general population of the GOLDN study. The majority of participants were re-recruited from three-generational pedigrees from two field centers in Minnesota and Utah. Nearly all individuals were of European descent. The study protocol has been published elsewhere [3], and was approved by the institutional review boards of the Universities of Alabama, Minnesota, and Utah and Tufts University.

Latent class analysis (LCA) was used to group all GOLDN individuals according to the diameter of their fasting (8-hour fast) VLDL, LDL and HDL particles, determined by nuclear resonance spectroscopy. LCA is a form of cluster analysis that groups individuals based on similarities within specified measures, and uses an iterative approach to determine the number of groups (classes) that best explains the population variance and covariance in the measures [4]. Initially, all participants are considered to be in a single class and classes are added until an additional class does not improve the fit of the model, as assessed using the log Bayes Factor (_log_*BF*). The LCA was implemented using the TRAJ Procedure in SAS [5]. As the LCA uses maximum likelihood estimation, the absolute diameter was standardized within each fraction of HDL, LDL and VLDL to ease optimization.

LCA grouped all GOLDN individuals into 8 classes (groups) according their fasting VLDL, LDL and HDL diameters. For two groups (N = 251), the average number of MetS features per member (3.5; Table 1) indicated that the majority of individuals within these groups met ATP-III criteria for the MetS. The two groups were not distinguished by the diameter of their LDL or HDL particles but by that of their VLDL particles (Table 1). The current analysis examined these groups for differences in three variants within the LPL gene.

### Genotype data

Genomic data was isolated from blood samples using routine DNA isolation sets (Qiagen, Valencia, CA, USA), using a TaqMan assay with allele-specific probes on the ABI Prism 7900HT Sequence Detection System (Applied Biosystems, Foster City, CA, USA) according to the standardized laboratory protocols.

### SNP selection

Three nonsynonymous cSNPs at *LPL *codons 9, 291, and 447, namely, D9N, M107 and S447X were chosen for literature reports of genetic associations or lipid-related biological function. The minor allele frequency (MAF) of M107 was 0, and so excluded from analysis.

### Statistical analysis

All analyses were conducted in SAS v9.2. Hardy-Weinberg equilibrium (HWE) was determined using *χ*^2 ^analysis. For genotype-phenotype associations, regression analysis was conducted using the "PROC GENMOD" command with genotype frequency, age, age^2 ^and age^3^, sex, and center of data collection as predictors, and LCA group as the outcome. Pedigree was treated as a fixed random effect. To increase power, carriers of the minor allele for each genotype were compared to non carriers.

## Results

### Participant characteristics

Lipoprotein, demographic and MetS-related characteristics are shown in Table 1.

### LPL genotypes

Genotype frequencies did not deviate from HWE expectations (*P *< .05). At the D9N locus, individuals were either homozygous for D9 allele (frequency = .94) or heterozygous (frequency = .06). The S447X locus showed individuals who were homozygous for the major allele (S447; frequency = .84), the minor allele (X447; frequency = .08) or heterozygous (frequency = .16).

### Genotype-phenotype associations

The D9N minor allele (MAF = .10) did not show a group association (P = .36; Table 1); however in the fully adjusted model (controlling for age, sex, data collection center and pedigree) there were significantly more carriers of the minor allele 447X variant (MAF = .08) in group 2, than group 1 (*P *= .04).

## Discussion

This study provides evidence that the minor allele of the *LPL *S774X variant is associated with larger VLDL diameters within groups defined by a lipoprotein diameter pattern which includes small LDL and HDL particles. The majority of individuals within these groups met ATP-III criteria for the MetS, and the pattern of large VLDL in addition to the small LDL and HDL particles additionally associates with higher IR, as well as the highest WC, fasting glucose and TGs, and diabetes prevalence. We did not find any association with lipoprotein diameter pattern and the D9N polymorphism.

The LPL S447X variant has been implicated in functional relevance to lipid parameters [6]. The main functions of the LPL S447X variant are the hydrolysis of plasma TGs, and in mediating the clearance of atherogenic remnant lipoproteins from the circulation [7]. Total missense, and nonsense mutations lead to type I hyperlipoproteinemia characterized by the accumulation of chylomicrons in the circulation [8]. The validity of our findings is emphazied by previous associations of the S447X variant with a number of lipid parameters [6,9,10], and also the presence of the MetS [10], and associations with peak LDL particle size [11]. However, this is the first study, to our knowledge, to associate variants with a pattern of particle sizes created using information from all three fractions of lipoprotein, which associates with the highest IR within the MetS.

The associations observed in this study must be viewed with a number of considerations. These include the small sample size combined with the low MAF for both variants, and the restriction of results to white Americans of European descent. It was this modest sample size that precluded gender stratified analyses. Nonetheless we provide evidence that the *LPL *447X allele associates with a pattern of particle sizes (small LDL and HDL, and large VLDL diameters) which, in turn, is associated with the higher IR, WC, fasting TG and glucose and diabetes prevalence among groups where individuals meet ATP-III criteria for the MetS. Given the small sample size, and novelty of the phenotype (lipoprotein diameter pattern) replication in additional studies is warranted.

## List of abbreviations

GOLDN: Genetics of Lipid-Lowering Drugs Network; HDL: High-density lipoprotein; LDL: Low-density lipoprotein; LPL: Lipoprotein Lipase; MetS: Metabolic syndrome; TG: Triglycerides; VLDL: Very low-density lipoprotein; WC: Waist circumference

## Competing interests

The authors declare that they have no competing interests.

## Authors' contributions

All authors read and approved the final manuscript. ACW carried out the analysis and wrote the manuscript, SG, WTG, EKK, IBB, HKT, MYT, PNH and JMO helped with critical revision of the manuscript, DKA is PI of the study and helped with critical revision of the manuscript.
